# Phosphatidylinositol 3-Kinase dependent upregulation of the epidermal growth factor receptor upon Flotillin-1 depletion in breast cancer cells

**DOI:** 10.1186/1471-2407-13-575

**Published:** 2013-12-05

**Authors:** Nina Kurrle, Wymke Ockenga, Melanie Meister, Frauke Völlner, Sina Kühne, Bincy A John, Antje Banning, Ritva Tikkanen

**Affiliations:** 1 Institute of Biochemistry, Medical Faculty, University of Giessen, Friedrichstrasse 24, 35392 Giessen, Germany

**Keywords:** Breast cancer, Signal transduction, Phosphatidylinositol kinase, Epidermal growth factor receptor, Oncogenes, Flotillin

## Abstract

**Background:**

Flotillin-1 and flotillin-2 are two homologous and ubiquitously expressed proteins that are involved in signal transduction and membrane trafficking. Recent studies have reported that flotillins promote breast cancer progression, thus making them interesting targets for breast cancer treatment. In the present study, we have investigated the underlying molecular mechanisms of flotillins in breast cancer.

**Methods:**

Human adenocarcinoma MCF7 breast cancer cells were stably depleted of flotillins by means of lentivirus mediated short hairpin RNAs. Western blotting, immunofluorescence and quantitative real-time PCR were used to analyze the expression of proteins of the epidermal growth factor receptor (EGFR) family. Western blotting was used to investigate the effect of EGFR stimulation or inhibition as well as phosphatidylinositol 3-kinase (PI3K) inhibition on mitogen activated protein kinase (MAPK) signaling. Rescue experiments were performed by stable transfection of RNA intereference resistant flotillin proteins.

**Results:**

We here show that stable knockdown of flotillin-1 in MCF7 cells resulted in upregulation of EGFR mRNA and protein expression and hyperactivation of MAPK signaling, whereas ErbB2 and ErbB3 expression were not affected. Treatment of the flotillin knockdown cells with an EGFR inhibitor reduced the MAPK signaling, demonstrating that the increased EGFR expression and activity is the cause of the increased signaling. Stable ectopic expression of flotillins in the knockdown cells reduced the increased EGFR expression, demonstrating a direct causal relationship between flotillin-1 expression and EGFR amount. Furthermore, the upregulation of EGFR was dependent on the PI3K signaling pathway which is constitutively active in MCF7 cells, and PI3K inhibition resulted in reduced EGFR expression.

**Conclusions:**

This study demonstrates that flotillins may not be suitable as cancer therapy targets in cells that carry certain other oncogenic mutations such as PI3K activating mutations, as unexpected effects are prone to emerge upon flotillin knockdown which may even facilitate cancer cell growth and proliferation.

## Background

Each year, hundreds of thousands of women around the world are diagnosed with breast cancer. Depending on the tumor stage upon diagnosis and the subtype of the cancer, the survival rates are highly variable. Although many treatment options are available, the best therapy depends on the molecular features of the tumor. For example, the so-called triple-negative tumors that lack estrogen and progesterone receptors and do not exhibit amplification/overexpression of the epidermal growth factor receptor (EGFR) family member ErbB2/Her2 cannot be treated with chemotherapeutic drugs that specifically target these molecules. Thus, personalized medicine, i.e. knowing the molecular signature of the tumor to be treated, has become essential for optimal and efficient treatment of cancers.

The phosphatidylinositol 3-kinase/protein kinase B (also known as AKT) signaling mode is an important regulator of cell survival, motility and growth for a review, see [1,2]. PI3 kinases (PI3K) can be activated by e.g. growth factor signaling and mediate the activation of AKT, a protein kinase with numerous substrates that include the mechanistic target of rapamycin (mTOR) and some members of the Forkhead transcription factor family, e.g. FOXO3 [3-6]. In line with its importance in cell survival, PI3K is frequently mutated in various tumors, especially in breast, gastric and colorectal cancers [7,8]. Most of the oncogenic mutations are found in the *PIK3CA* gene (GenBank: NM_006218.2) that encodes for the catalytic p110α subunit of PI3K. The most frequently observed mutations in this protein in cancers are the H107R substitution in the kinase domain and E545K in the helical domain [8-10]. Both mutation result in constitutive activation of PI3K/AKT signaling and contribute to cellular transformation [11,12].

Flotillin-1 and flotillin-2 are highly conserved proteins that are associated with specific lipid microdomains in cellular membranes [for a review, see [13,14]. Flotillins reside on the cytoplasmic face of membranes [15] and exhibit a broad cell type and stimulus dependent cellular localization. In many cells, flotillins are found at the plasma membrane and endosomal structures, but they have also been shown to localize to the nucleus, cell-matrix adhesions, the Golgi and phagosomes [16-21]. Flotillins have been suggested to function in membrane trafficking processes such as endocytosis and recycling, in cell-matrix and cell-cell adhesion but also in receptor tyrosine kinase signaling [17,19,20,22-31]. We have recently shown that flotillin-1 is important for the proper activation and clustering of the EGFR after ligand binding. Furthermore, downstream signaling from EGFR towards the mitogen activated protein kinase (MAPK) cascade requires flotillin-1 which can directly interact with the proteins of the MAPK cascade and functions as a novel MAPK scaffolding protein [16], reviewed in [32]. During EGFR signaling, flotillins are Tyr phosphorylated by the Src family kinases and become endocytosed from the plasma membrane into endosomes [17,27]. However, they do not appear to be involved in EGFR endocytosis [16].

Several studies have shown that flotillins are important regulators of cellular signaling and their overexpression is associated with various types of cancers, such as melanoma, breast cancer, head and neck cancer and gastric cancer [29,33-37]. Importantly, flotillin overexpression was shown to correlate with poor prognosis and shorter survival of the patients. First findings suggesting a potential connection of flotillins with cancer were published almost a decade ago when Hazarika *et al.* showed that flotillin-2 overexpression is associated with metastatic potential in melanoma [34]. In gastric cancer, flotillin-2 levels show a correlation with Her2 expression and are associated with poor prognosis [37], whereas in head and neck cancer, flotillin-2 overexpression shows a strong predictive value for the development of metastases [36]. In breast cancer, increased flotillin-2 levels correlate with reduced patient survival [29].

Due to the above findings and importance of flotillins for signaling pathways that regulate cell proliferation, it has been suggested that flotillins may represent promising targets for cancer therapy. In line with this, acute flotillin depletion impairs signaling and cell proliferation in some cancer cells, as shown by us and others [16,29,35], and flotillin deficiency in a mouse breast cancer model reduces the formation of metastases [33]. We here show that stable knockdown of flotillin-1 in the human breast adenocarcinoma MCF7 cell line results in upregulation of EGFR mRNA and protein expression and hyperactivation of MAPK signaling, whereas ErbB2 and ErbB3 expression are not affected. We provide evidence that the overexpression of EGFR in MCF7 cells is dependent on the activity of phosphatidylinositol 3-kinase (PI3K) which carries the E545K activating mutation in the catalytic subunit of PI3K. Thus, this study demonstrates that great caution is required when flotillin expression is targeted in cancer cells, as unexpected effects may emerge that even facilitate cancer cell growth and proliferation.

## Methods

### Antibodies

Rabbit polyclonal antibody against EGFR (D38B1) and antibody against phospho-EGFR (pTyr1173), AKT, AKT2 (5B5), phospho-AKT (Ser473), MEK1/2, phospho-MEK1/2 (Ser217/221) and phospho-Raf1 (pSer338) were purchased from Cell Signaling Technology (Danvers, MA, USA). Rabbit polyclonal antibodies against ERK2 and Raf-1 and mouse monoclonal antibodies against phospho-ERK1/2 (Tyr204), LAMP3/CD63 and EGFR (528) were purchased from Santa Cruz Biotechnology (Santa Cruz, CA, USA). A mouse monoclonal antibody against GAPDH was from Abcam. Rabbit polyclonal antibodies against flotillin-1 and flotillin-2 were purchased from Sigma-Aldrich (Taufkirchen, Germany). For detection of E-cadherin, flotillin-1 or flotillin-2 in Western blots, monoclonal mouse antibodies from BD Transduction Laboratories (Franklin Lakes, NJ, USA) were used. For enhancing the GFP signal in rescue experiments we used a polyclonal GFP antibody (Clontech Laboratories, Inc., Takara Bio Group). The primary antibodies used for immunofluorescence were detected with a Cy3 conjugated goat anti-mouse antibody (Jackson ImmunoResearch, West Grove, PA, USA) and with an Alexa Fluor 488 donkey anti-rabbit antibody (Life Technologies, Karlsruhe, Germany). The primary antibodies used for Western blotting were detected with a HRP conjugated goat anti-mouse or goat anti-rabbit antibody (Dako, Glostrup, Denmark).

### Cell culture and RNA interference

MCF7 cells were cultured in Dulbecco’s Modified Eagle’s Medium (DMEM high glucose) supplemented with 10% fetal bovine serum (Life technologies) and 1% penicillin/streptomycin at 37°C under 5% CO_2_. Expression of flotillin-1 and flotillin-2 was stably knocked down in MCF7 cells using the Mission Lentiviral *sh*RNA system (Sigma-Aldrich), with two viruses each targeting different sequences in human flotillin-1 or flotillin-2. The control cells were established using an *sh*RNA that does not target any human gene. Establishment of the stable knockdown cell lines was done as described previously for HeLa cells [17].

### Plasmids, transfection and generation of stable MCF7 cells

Full length human flotillin-1-pEGFP was a kind gift of Duncan Browman. For the generation of RNAi resistant flotillin-1-pEGFP constructs, mutagenesis was carried out with the QuikChange Site-Directed Mutagenesis Kit (Stratagene, La Jolla, USA) according to the manufacturer’s protocol using the primers listed in Table 1. Rat-flotillin-2-EGFP [26], which is resistant against the human *sh*RNA sequences due to natural silent substitutions in the rat sequence, was used for flotillin-2 rescue experiments. For stable plasmid transfections of MCF7 knockdown cells, we used the Neon electroporation system (Life Technologies) with following settings: 400,000 cells, 1230 V, 20 mV, 5 μg plasmid DNA. After transfection, stable clones were selected for six weeks with G418 (500 μg/ml).

**Table 1:** Primers used in this study

**Primer Name**	**Sequence**
Rpl13a forward	5^′^-CCTGGAGGAGAAGAGGAAAGAGA-3^′^
Rpl13a reverse	5^′^-TTGAGGACCTCTGTGTATTTGTCAA-3^′^
GAPDH forward	5^′^-CATCTTCCAGGAGCGAGATCCC-3^′^
GAPDH reverse	5^′^-CCAGCCTTCTCCATGGTGGT-3^′^
EGFR-A for	5^′^-AAAGAAAGTTTGCCAAGGCACGA-3^′^
EGFR-A rev	5^′^-CTCCACTGTGTTGAGGGCAATGAG-3^′^
EGFR-B for	5^′^-ATCTGCCTCACCTCCACCGT-3^′^
EGFR-B rev	5^′^-CCAAGTAGTTCATGCCCTTTGCGA-3^′^
Cyclin D1 for	5^′^-TCGTGGCCTCTAAGATGAAGGA-3^′^
Cyclin D1 rev	5^′^-CAGCTCCATTTGCAGCAGCTC-3^′^
Flot1-RNAi-res-A for	5^′^-CACACTGACCCTAAACGTCAAGAGCGAGAAGGTTT ACACTC-3^′^
Flot1-RNAi-res-A rev	5^′^-GAGTGTAAACCTTCTCGCTCTTGACGTTTAGGGTCA GTGTG-3^′^
Flot1-RNAi-res-B for	5^′^-CTAGCCGAGGCCGAGAAATCCCAGCTAATTATGCA GGC-3^′^
Flot1-RNAi-res-B rev	5^′^-GCCTGCATAATTAGCTGGGATTTCTCGGCCTCGGCT AG-3^′^

### Growth factor and inhibitor treatment

MCF7 cells were serum-starved for 16 hours before treatment with 100 ng/ml epidermal growth factor (EGF, Sigma-Aldrich) for the indicated times. For the inhibition of EGFR tyrosine kinase, MCF7 cells were serum-starved for 20 hours and treated with 1 μM AG9 (control) or 1 μM PD153035 (EGFR kinase inhibitor) for 5 min at 37°C prior to stimulation with 100 ng/ml EGF for 10 min at 37°C. For PI3 kinase inhibition, MCF7 cells were treated in normal growth medium with 20 μM Ly294002 (PI3K inhibitor) or DMSO (control) for 24 hours at 37°C.

### Immunofluorescence

Cells were cultured on coverslips and fixed with methanol at −20°C. The cells were labeled with primary antibodies and Cy3 and/or Alexa Fluor488 conjugated secondary antibodies and then embedded in Gel Mount (Biomeda, Foster City, USA) supplemented with 1,4-diazadicyclo(2,2,2)-octane (50 mg/ml; Fluka, Neu-Ulm, Germany). The samples were analyzed with a Zeiss LSM710 Confocal Laser Scanning Microscope (Carl Zeiss, Jena, Germany).

### Cell lysis, gel electrophoresis and Western blot

Cell pellets were lysed in lysis buffer (50 mM Tris–HCl pH 7.4, 150 mM NaCl, 2 mM EDTA, 1% Nonidet P-40) supplemented with protease inhibitor cocktail (Sigma-Aldrich), 1 mM sodium fluoride and 1 mM sodium orthovanadate (for EGF stimulation) and lysates were cleared by centrifugation. Protein concentration was measured with the Bio-Rad protein assay reagent (Biorad, Munich, Germany). Equal protein amounts of the lysates were analyzed by SDS-PAGE and Western blot.

### RNA isolation and quantitative PCR

RNA was isolated using the NucleoSpin RNA purification kit (Macherey-Nagel, Düren, Germany). Of each MCF7 clone, 3 μg of RNA was reverse-transcribed with 2 μM oligo(dT) primers, 2 μM random primers (NEB) and 200 units Moloney murine leukemia virus reverse transcriptase (ProtoScript II reverse transcriptase, NEB) in a total volume of 20 μl. Real-time PCRs (CFX connect 96 – QPCR-System, Bio-Rad) were performed in duplicates with 0.5 μl of 5-fold diluted cDNA in a 13 μl reaction using SensiFAST SYBR NoROX-Kit (Bioline, Luckenwalde, Germany). The annealing temperature was 66°C for all PCR reactions. Primers were designed to be specific for cDNA with PerlPrimer (Table 1). The mean of the reference genes *Rpl13a* and *GAPDH* was used for normalization.

### Cell viability assay

MCF-7 cells were seeded in 12-well plates at an initial density of 5 × 10^5^ cells/well. The following day, they were treated with 3-[4,5-dimethylthiazol-2-yl]-2,5-diphenyl tetrazolium bromide (0.5 mg/ml, Sigma-Aldrich) at 37°C for 2–4 hours. Thereafter, 600 μl DMSO was added to the cells to dissolve the formazan crystals, and the absorbance was measured at 570 nm, with reference at 690 nm.

### Statistical analysis

Unless otherwise stated, all experiments were performed at least three times. For the statistical analysis, Western blot bands of proteins were quantified by scanning densitometry using Quantity One Soft-ware (Bio-Rad) and normalized to GAPDH or as indicated. Phosphorylated proteins were normalized against the total amount of the respective protein. Data are shown as the mean ± SD. Statistical comparisons between groups were made using one-way or two-way analysis of variance (ANOVA) as appropriate using GraphPad Prism 6 software. Values of *p* < 0.05 were considered significant (*), whereas values of *p* < 0.01 and *p* < 0.001 were defined very significant (**) and highly significant (***), respectively.

### Electronic manipulation of images

The images shown have in some cases as a whole been subjected to contrast or brightness adjustments. No other manipulations have been performed unless otherwise stated.

## Results

### Generation of stable knockdown MCF7 cell lines for flotillins

**Figure 1:**
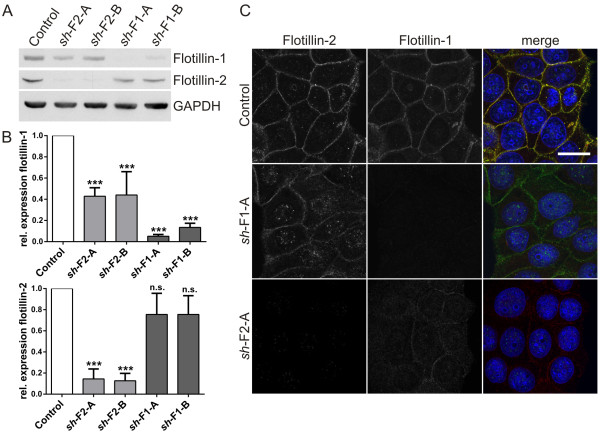
**Characterization of MCF7 cells stably depleted of flotillin-1 and flotillin-2. (A)** Expression of flotillins in MCF7 cells depleted of flotillin-1 (*sh*-F1-A/B) or flotillin-2 (*sh*-F2-A/B). GAPDH was detected to show equal loading. **(B)** Densitometric quantification of flotillin-1 and flotillin-2. The signals were normalized to GAPDH. Bars represent the mean ± SD of three independent experiments. Statistical analysis using one-way ANOVA. ***, p < 0.001. **(C)** Staining of endogenous flotillin-1 and flotillin-2 in MCF7 cells depleted of flotillin-1 (*sh*-F1-A) or flotillin-2 (*sh*-F2-A). Scale bar: 20 μm.

Flotillins have been previously connected to various cancers, including breast cancer. To study the function of flotillins in breast cancer cells, we generated human MCF7 cell lines in which flotillin-1 or flotillin-2 expression was stably knocked down by means of lentivirus mediated short hairpin RNAs (*sh*RNAs). The knockdown cell lines showed a profound reduction of the respective flotillin protein (85-95%), as detected by means of Western blot (Figure 1A-B). Although in most cell lines we have studied so far, flotillin-2 knockdown results in destabilization and depletion of flotillin-1 protein as well, we detected substantial amounts of flotillin-1 (>40% of the control) in flotillin-2 knockdown cells. However, flotillin-2 amount was unchanged in in flotillin-1 knockdown cells. These results were further corroborated by means of immunostaining (Figure 1C) which showed results consistent with the Western blot analysis. Staining for the other two flotillin knockdown cell lines are shown in Additional file 1A. Consistent with the findings of Lin *et al.* in MCF7 cells, flotillin knockdown resulted in a mild impairment of viability (Additional file 1B).

### Expression of the EGF receptor is increased in flotillin-1 knockdown cells

**Figure 2:**
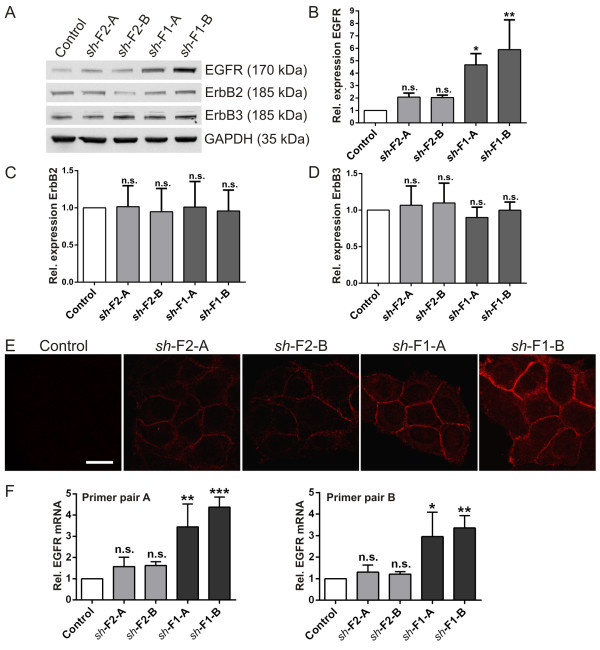
**Expression of the epidermal growth factor receptor family members in MCF7 cells depleted of flotillin-1 or flotillin-2.** Expression of the epidermal growth factor receptor family members EGFR, ErbB2 and ErbB3 in MCF7 cells depleted of flotillin-1 (*sh*-F1-A/B) or flotillin-2 (*sh*-F2-A/B). **(A)** Western blot using specific antibodies. GAPDH was detected to show equal loading. **(B-D)** Densitometric quantification. The signals were normalized to GAPDH. Bars represent the mean ± SD of three independent experiments. Statistical analysis using one-way ANOVA. *, p < 0.05; **, p < 0.01. **(E)** Staining of endogenous EGFR in MCF7 cells depleted of flotillin-1 (*sh*-F1-A/B) or flotillin-2 (*sh*-F2-A/B). Scale bar: 20 μm. **(F)** Quantitative real-time PCR analysis showing the relative mRNA level of EGFR in MCF7 control and flotillin-1 (*sh*-F1-A/B) or flotillin-2 (*sh*-F2-A/B) knockdown cells using two different primer pairs. The expression was normalized to GAPDH and Rpl13a. Bars represent the mean ± SD of three independent experiments. Statistical analysis using one-way ANOVA. *, p < 0.05; **, p < 0.01; ***, p < 0.001.

Breast cancer cells frequently exhibit an increased amount of the HER2/ErbB2 receptor protein that belongs to the EGFR receptor family. Recent data have shown that in gastric tumors, flotillin-2 expression correlates with HER2/ErbB2 levels and flotillin-2 knockdown in a gastric cancer cell line results in reduced HER2 expression [37]. Our recent data suggest that EGFR signaling is impaired upon flotillin-1 knockdown in HeLa cells [16]. Thus, we measured the expression of EGFR, ErbB2 and ErbB3 in our stable knockdown MCF7 cells (Figure 2). Surprisingly, the expression of EGFR was significantly increased in flotillin-1 knockdown cells (Figure 2A and B), whereas neither ErbB2 (Figure 2C) nor ErbB3 (Figure 2D) exhibited an altered expression. Flotillin-2 knockdown cells showed a mildly but not significantly increased EGFR expression, consistent with the partial reduction of flotillin-1 in these cells. The increase in EGFR expression was also clearly detectable by means of immunofluorescence (Figure 2E). Although EGFR was virtually undetectable in control *sh*RNA MCF7 cells by antibody staining, we readily observed a plasma membrane associated staining in all flotillin knockdown cells, consistent with the increased expression (Figure 2E).

In breast cancer, EGFR overexpression is mainly based on transcriptional regulation [38]. To study if the increased EGFR expression is mediated by transcriptional upregulation or reduced protein turnover, we measured the mRNA of EGFR by means of quantitative real-time PCR with two different primer pairs (Figure 2F). In line with the higher protein amount, EGFR mRNA was significantly increased in flotillin-1 knockdown cells, whereas flotillin-2 knockdown cells exhibited a tendency to a higher EGFR mRNA, which did not reach significance (Figure 2F).

### EGF induced endocytosis of EGFR is not impaired in flotillin-1 knockdown cells

**Figure 3:**
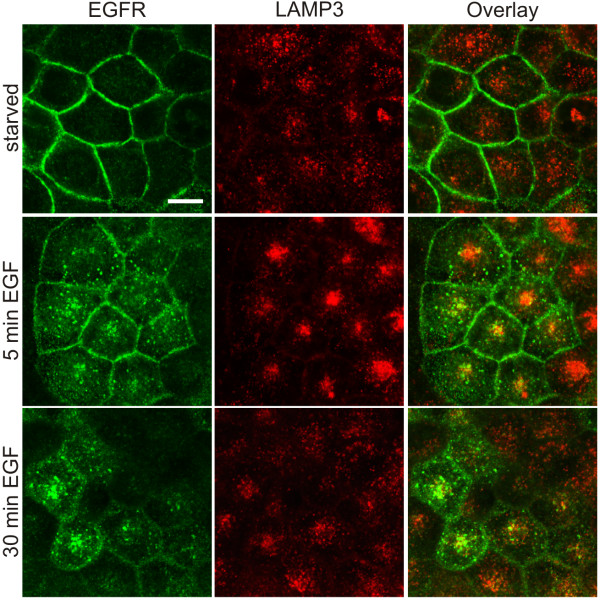
**EGFR endocytosis is not impaired upon flotillin-1 depletion.** Staining of endogenous EGFR (green) and LAMP3 (red) after EGF-stimulation (100 ng/ml) for 5 and 30 minutes in MCF7 cells depleted of flotillin-1 (*sh*-F1-B). Scale bar: 20 μm.

Flotillin-1 has been suggested to be involved in the endocytosis of various proteins [19,23,39]. Since inhibition of EGFR endocytosis might affect its half-life and thus contribute to the increased amount seen in flotillin-1 knockdown cells, we checked by means of immunofluorescence staining if EGFR endocytosis was impaired. These experiments were only performed in flotillin-1 knockdown cells, as EGFR staining was not visible in the control cells due to its low expression level (Figure 2E). Rapid endocytosis of EGFR was found to occur despite flotillin-1 depletion. Already after 5 min of EGF stimulation, EGFR was detected in perinuclear vesicular structures where it colocalized with LAMP3/CD63, which is a marker for multivesicular bodies and late endosomes. The amount of the endocytosed receptor increased upon 30 min of stimulation. However, the staining pattern was slightly different from that observed after 5 min of EGF, and EGFR became less concentrated in the perinuclear region but still colocalized with LAMP3 in more peripheral vesicular structures (Figure 3). Thus, flotillin-1 depletion does not appear to inhibit EGFR endocytosis from the plasma membrane, consistent with our prior findings in HeLa cells [16].

### EGFR expression can be reduced upon flotillin re-expression

**Figure 4:**
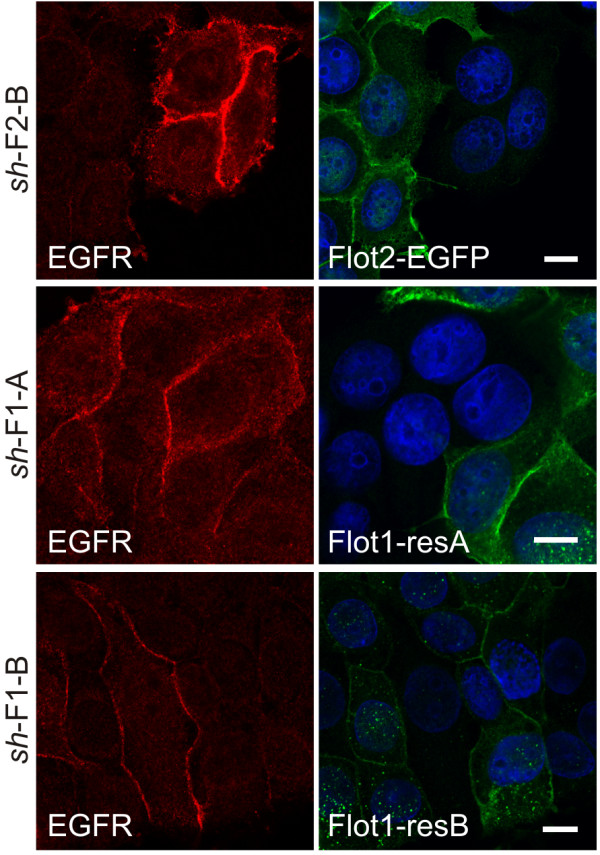
**Ectopic expression of flotillins normalizes EGFR expression in stable MCF7 flotillin knockdown cells.** MCF7 control and flotillin-1 (*sh*-F1-A/B) or flotillin-2 (*sh*-F2-B) knockdown cells stably transfected with flotillin-2 (Flot2-EGFP) and flotillin-1 (Flot1-resA/-resB) constructs were grown subconfluent on coverslips, fixed and stained with antibodies against endogenous EGFR (red) and GFP (green). EGFP was stained with antibodies to enhance the signal as the expression levels in the stable cells were kept low in order not to exceed that of endogenous flotillins in normal cells. Nuclei were stained with DAPI. Scale bar: 10 μm.

To show a direct causative connection between flotillin depletion and EGFR expression levels, we performed rescue experiments by stably re-expressing EGFP-tagged flotillins in the knockdown cells. For this purpose, rat flotillin-2-EGFP [26] which is identical to the human one at protein level but distinct at the DNA level, resulting in resistance against the *sh*RNAs, was used. For flotillin-1, we used a human flotillin-1-EGFP construct that was converted resistant towards the *sh*RNAs by targeted silent mutations. The increased EGFR amount in flotillin knockdown cells was indeed reduced upon re-expression of the respective flotillin in these cells (Figure 4). Since not all of the cells shown express the rescue constructs, they provide an internal control, and the reduction of EGFR amount was only seen in cells re-expressing flotillins. Thus, these data show that the increased EGFR expression in the flotillin knockdown MCF7 cells is a direct consequence of flotillin depletion.

### EGFR induced signaling towards MAP kinases is increased in flotillin knockdown cells

**Figure 5:**
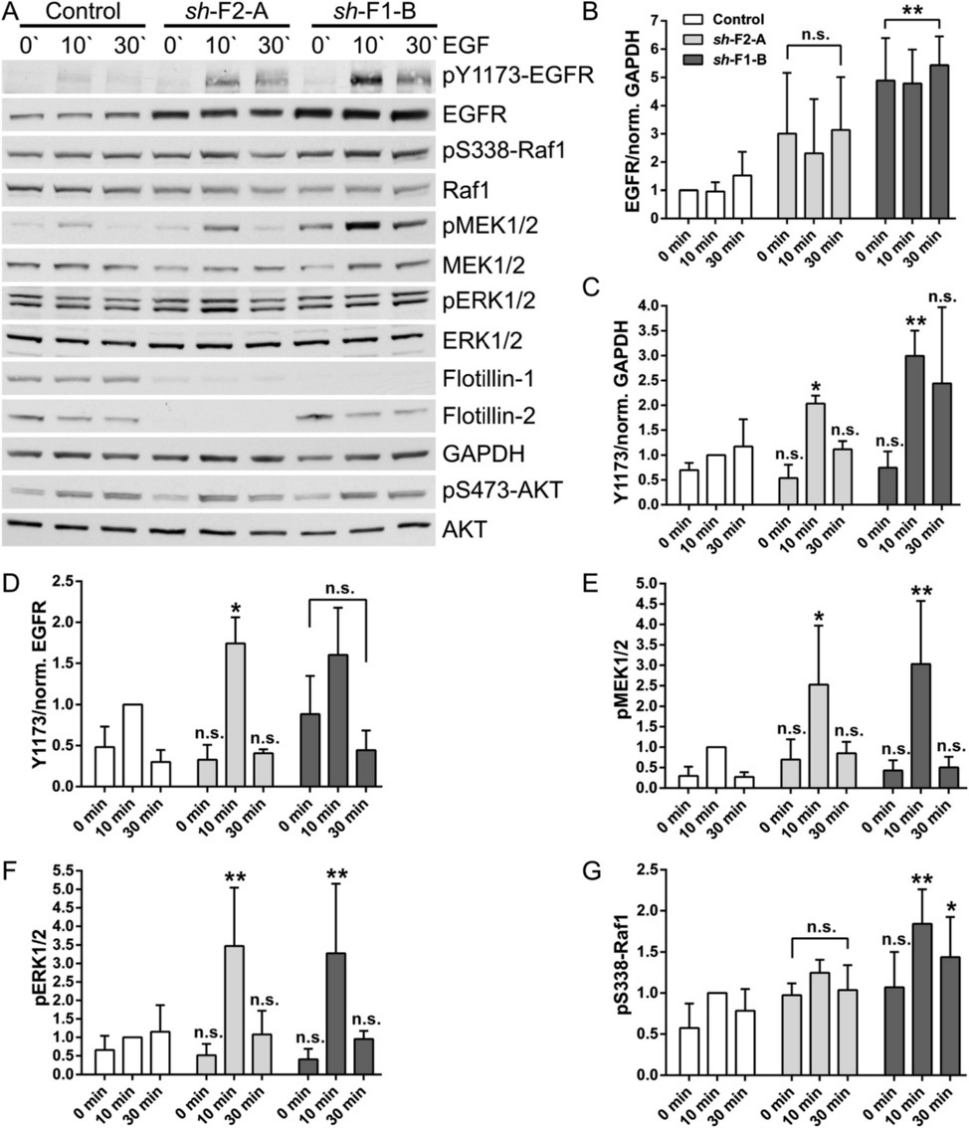
**EGF stimulation of MCF7 cells depleted of flotillin-1 or flotillin-2. (A)** Western blot with specific antibodies for pY1173-EGFR, EGFR, pS473-AKT, AKT, pS338-Raf1, Raf1, pMEK1/2, MEK1/2, pERK1/2, ERK1/2, flotillin-1, flotillin-2 and GAPDH in MCF7 cells depleted of flotillin-1 (*sh*-F1-B) or flotillin-2 (*sh*-F2-A) after EGF-stimulation (100 ng/ml) for 10 and 30 minutes. **(B-G)** Densitometric quantification of EGFR **(B)**, pY1173-EGFR **(C, D)**, pMEK1/2 **(E)**, pERK1/2 **(F)** and pS338-Raf1 **(G)**. The signals for total protein were normalized to GAPDH, phosphorylated proteins to the corresponding total protein. Bars represent the mean ± SD of four independent experiments. Statistical analysis using two-way ANOVA. *, p < 0.05; **, p < 0.01.

To show that the increase in EGFR amount also culminates in an increased downstream signaling response, we stimulated the cells with EGF for 10 and 30 min after overnight serum starvation. The activation of the MAP kinase cascade was detected by Western blot by means of antibodies specific to the activated kinases of this pathway. Figure 5 shows the respective blots (Figure 5A) together with the quantification data (Figure 5B-G). The data for the further two cell lines are shown in Additional file 2. Consistent with the above data, the flotillin-1 knockdown cells showed a significantly increased EGFR expression (Figure 5B). The phosphorylation of EGFR in Tyr1173 (pY1173), when normalized to GAPDH, showed a significant increase upon 10 min of stimulation in all four knockdown cell lines (Figure 5C). When the phosphorylation was correlated to the amount of EGFR, these values barely reached significance (Figure 5D), implicating that the receptors are activated to a normal degree, and the increased pY1173 is due to increased receptor amount. Phosphorylation of both MEK1/2 and ERK1/2 were also significantly increased after 10 min EGF in flotillin-1 knockdown cell lines (Figure 5E-F, Additional file 2), whereas the amount of total MEK and ERK was not changed (data not shown). Phosphorylation of Raf-1 at Ser338 was significantly increased in one of the flotillin-1 knockdown lines (Figure 5G). Consistent with the upregulation of MAP kinase signaling, we found that the mRNA for the downstream target cyclin D was increased in flotillin-1 knockdown cells (Additional file 1C). We also detected the activation of protein kinase B/AKT in our knockdown cells (Figure 5A). Although the signal for phospho-Ser473 of AKT tended to be higher in flotillin knockdown cells, it only reached significance at 10 min EGF stimulation in one of the flotillin-2 knockdown clones (data not shown). This is most likely due to the fact that MCF7 cells exhibit a constitutively active PI3 kinase which causes a relatively high basal AKT activity (as seen in the starved cells in Figure 5A). No change in the amount of total AKT was detected. Taken together, these data show that the increased EGFR in flotillin knockdown cells is signaling compatible and enhances MAP kinase signaling in these cells.

**Figure 6:**
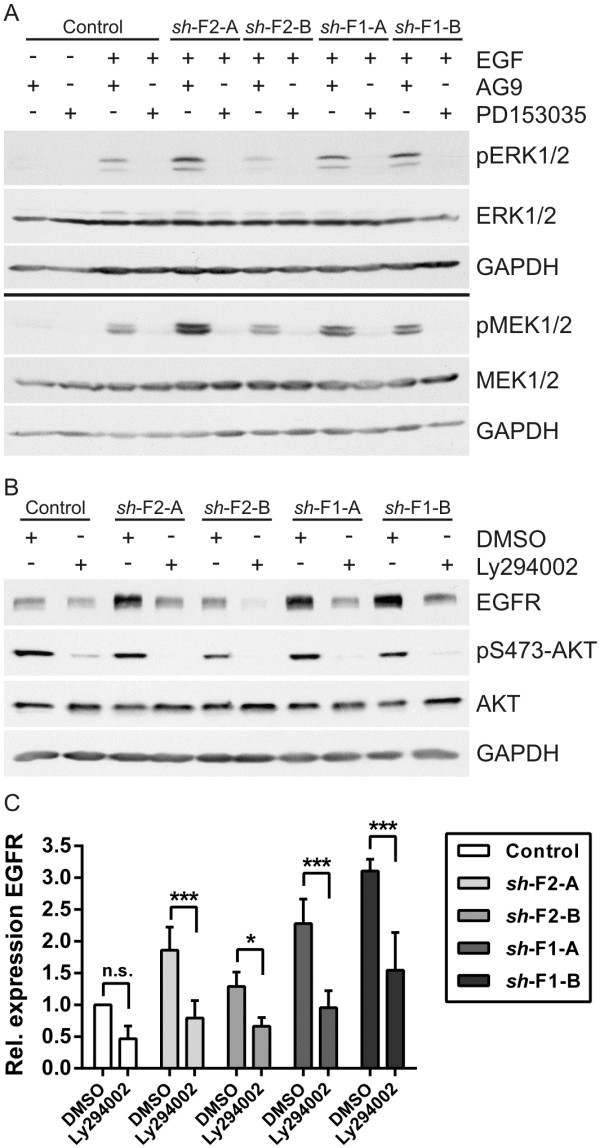
**Upregulation of EGFR in flotillin depleted MCF7 cells is PI3K-dependent. (A)** MCF7 control and flotillin-1 (*sh*-F1-A/B) or flotillin-2 (*sh*-F2-A/B) knockdown cells were treated with 1 μM EGFR kinase inhibitor (PD153035) and 1 μM AG9 (control) for 5 minutes prior to stimulation with EGF (100 ng/ml). Western blot with specific antibodies was used to detect pERK1/2, ERK1/2, pMEK1/2, MEK1/2. Loading control: GAPDH. **(B)** The cells were treated with 20 μM PI3K inhibitor (Ly294002) and DMSO (control) for 24 hours, followed by detection of EGFR, pS473-AKT and AKT. Loading control: GAPDH. **(C)** Densitometric quantification of EGFR. The signals were normalized to GAPDH. Bars represent the mean ± SD of four independent experiments. Statistical analysis using two-way ANOVA. *, p < 0.05; ***, p < 0.001.

To show that the increased MAPK signaling is due to EGFR activity and not activation of some other signaling pathway, we used PD153035, an EGFR kinase inhibitor. AG9, a non-inhibiting compound was used as a control. Treatment of the cells with the EGFR inhibitor resulted in a profound inhibition of both ERK and MEK in EGF stimulated control and flotillin knockdown cells (Figure 6A). Thus, increased EGFR activity due to its overexpression is responsible for the increase in MAPK signaling upon flotillin knockdown.

### Constitutive activity of PI3K causes EGFR overexpression upon flotillin knockdown

MCF7 cells exhibit a constitutively active PI3K due to an E545K activating mutation in the gene encoding for the catalytic subunit of the PI3K [40]. Since EGFR may be transcriptionally regulated by PI3K signaling, and we have not observed a similar upregulation of EGFR in other cell lines upon flotillin knockdown, we tested if PI3K inhibition would be sufficient to return EGFR expression back to the level of control cells. For this, MCF7 cells were incubated with the PI3K inhibitor Ly294002 for 24 hours under normal culturing conditions. Inhibition of PI3K was verified by checking AKT phosphorylation which was almost completely inhibited upon PI3K inhibitor treatment. Intriguingly, PI3K inhibition resulted in very profound reduction in EGFR levels in flotillin knockdown cells, whereas it showed a much lower effect in the control cells (Figure 6B). Quantification of the data showed a statistically significant reduction of EGFR expression upon PI3K inhibition on the protein level (Figure 6C), whereas the mRNA levels of EGFR were not significantly reduced (Additional file 3). These data suggest that upon loss of flotillin-1, the constitutively active PI3K induces the upregulation of EGFR protein expression in MCF7 cells.

## Discussion

We have here used the human breast adenocarcinoma MCF7 cell line to study the role of flotillins in breast cancer signaling. Previous studies have suggested that flotillin ablation might be a promising therapy option in tumors that exhibit flotillin overexpression [29,33,35,37]. However, we here show that decreased flotillin-1 expression may result in a paradoxical increase in signaling due to upregulation of receptors functionally connected to flotillins. Although most studies on flotillins in cancer have described an elevated flotillin-2 expression, most of them did not address flotillin-1 directly [34,36,37] or found that flotillin-1 expression has no predictive value in terms of e.g. patient survival [29]. However, flotillins are strongly interdependent in most cells, as shown by us and others, and even in the flotillin-1 [25] and flotillin-2 [33,41] knockout mice. Generally, flotillin-1 shows a higher dependency on flotillin-2 expression, so that flotillin-2 depletion results in profound reduction of flotillin-1 expression, whereas the effect of flotillin-1 ablation on flotillin-2 levels is less pronounced. Although it is not clear if flotillin-2 overexpression in tumors also results in elevated flotillin-1 expression, it would be important to clarify this issue as flotillins may not be functionally identical.

In the MCF7 cells used in our study, the interdependency of flotillins appears to be less strong, and considerable amounts of flotillin-1 (about 40%) are still expressed in the absence of flotillin-2. Importantly, EGFR overexpression and increase in signaling correlated with flotillin-1 amount, and cells depleted of flotillin-2 showed a weaker effect, suggesting that the upregulation of EGFR is directly dependent on the flotillin-1, but not flotillin-2, amount. These data are well in agreement with our previous findings showing that flotillin-1 is involved in EGFR activation and MAPK signaling [16].

We here discovered a specific upregulation of EGFR upon flotillin-1 ablation, whereas no change in the levels of ErbB2 or ErbB3 was detected. EGFR was transcriptionally elevated in the absence of flotillin-1, which is the main regulatory mechanism of EGFR in most tumors showing increased EGFR expression [38]. Thus, reduced degradation alone is unlikely to be responsible for the elevated EGFR expression in MCF7 cells, since rapid endocytosis of EGFR upon EGF stimulation took place despite flotillin-1 ablation. Unfortunately, it was not possible to measure EGFR recycling, the elevation of which might also result in slower receptor degradation and increased amount, as these experiments would require a comparison to the control cells which show too low expression of EGFR for direct comparisons.

EGFR expression has been shown to be regulated by many factors that regulate growth and proliferation. In breast cancer, EGFR and ErbB2 expression was found to be under control of the Y-box transcription/translation factor YB1 which is phosphorylated by Akt [42,43]. However, YB1 has been shown to regulate both EGFR and ErbB2 expression [42,44]. As we did not observe upregulation of ErbB2 in our flotillin-1 knockdown cells, YB1 is not very likely to be the cause of EGFR upregulation upon flotillin-1 knockdown.

Interestingly, previous studies have suggested that elevated flotillin-2 expression in gastric cancers correlates with ErbB2 levels [37], and flotillins are required to stabilize ErbB2 in the plasma membrane in SKBR3 breast cancer cells [29]. Depletion of either of the flotillin proteins resulted in increased endocytosis and degradation of ErbB2 in these cells, implicating that flotillins regulate ErbB2 trafficking. Furthermore, flotillins were found to form complexes with ErbB2, which also contained the heat shock protein Hsp90 [29]. However, this appears not to be the case in MCF7 cells in which the amount of ErbB2 was not altered upon flotillin depletion. Thus, it is evident that flotillins exhibit different effects on receptor trafficking and signaling in breast cancer cells of different origin. This is not surprising, considering that the cell lines used are different in terms of their genetic background and oncogenic mutations that are present in these cells. For example, according to the Sanger institute COSMIC database [40], MCF7 cells exhibit a mutation in the catalytic subunit of PI3K, whereas SKBR3 cells have a WT PI3K. However, both cell lines express non-mutated EGFR and Ras proteins.

Another factor that might affect the results obtained in various studies is the means of knocking down flotillin expression. For example, Lin *et al.*[35] described that flotillin-1 knockdown in MCF7 cells reduces cell viability and impairs tumorigenicity in MCF7 cells. In contrast to these data, we here observed elevated MAPK signaling and increased cyclin D mRNA expression upon flotillin-1 ablation. Furthermore, Lin *et al.* detected a decreased AKT phosphorylation and concomitant upregulation of the forkhead transcription factor Foxo3 which is associated with decreased cell viability due to upregulation of apoptotic genes. Although Foxo3 expression was increased in our flotillin-1 knockdown cells (data not shown), we did not observe any evident impairment of AKT activation (see Figure 6B), in contrast to Lin *et al*. Since AKT activity negatively affects Foxo3 function by means of a direct phosphorylation, it is plausible that the increased Foxo3 expression in flotillin knockdown cells is compensated by the normal AKT activity, thus preventing Foxo3 from increasing cell death in these cells. Furthermore, PI3K mutations have been shown to promote resistance against apoptosis [11,45] and may thus protect against increased Foxo3 activity.

There is one significant difference in the experimental setting as compared to our study. Lin *et al.* apparently used a short-term, acute knockdown of flotillins [35], whereas we have here generated stable flotillin knockdown MCF7 cell lines. We think that the stable knockdowns are more representative of the situation in tumors, as adaptation to flotillin deficiency may result in compensatory upregulation of signaling proteins, as shown in the present study, which may not be possible upon acute knockdown. In line with this, Berger *et al.* recently showed that although flotillin-2 deficiency in a mouse breast cancer model caused a reduced lung metastasis formation, it showed no effect on the growth of primary tumors [33]. Similarly, we have detected an upregulation of MAPK signaling and expression of several growth associated genes in various organs of our flotillin-2 knockout mouse model generated independently of that of Berger *et al.*[41]. Thus, long term effects of flotillin ablation may be unpredictable due to compensatory mechanisms, especially in cancer patients.

We have so far only observed the upregulation of EGFR in MCF7 cells upon stable flotillin depletion. Since MCF7 cells display a constitutively active PI3K due to the E545K mutation [40], this prompted us to study if increased PI3K signaling might be the cause of EGFR upregulation upon flotillin-1 silencing. Indeed, EGFR amount was efficiently downregulated upon inhibition of PI3K activity. EGFR is not upregulated e.g. in human breast epithelial MCF10A, cervix carcinoma HeLa or human keratinocyte HaCat cells upon stable flotillin-1 knockdown (our unpublished findings). Expression of flotillins in these cells lines is not much different from MCF7 cells, but they all exhibit a WT PI3K [40]. This may suggest that flotillins are required to keep EGFR amount under control when PI3K is constitutively activated. This is very likely to occur at least in part by means of increased activation of an as yet unidentified transcription factor that regulates EGFR transcription (see also above) and whose activation also depends on PI3K signaling. Since activating PI3K mutations that are oncogenic [11,12] are present in about 25% of breast tumors [7-9], and E545K is one of the most common PI3K mutations in breast cancer, it will be of uttermost importance to clarify the mutation status of breast cancer patients before aiming at treatments based on flotillin ablation.

## Conclusions

Due to recent findings showing flotillin overexpression in various cancer types, flotillins have been suggested to be promising cancer therapy targets. This idea is also supported by the fact that genetic ablation of flotillins in the mouse is well tolerated. However, we here show that flotillin depletion may result in unexpected hyperactivation of proliferative signaling pathways, depending on the molecular signature of the tumor. Thus, before cancer therapies based on functional impairment of flotillins are developed, it will be important to clarify the crosstalk between flotillins and oncogenic mutations that are frequently found in specific cancers.

## Competing interests

The authors declare that they have no competing interests.

## Authors’ contributions

Experiments were performed by NK, WO, MM, FV, SK, BJ and AB. RT generated the stable flotillin knockdown cell lines. NK made the figure drafts, RT the final versions. NK and RT wrote the manuscript. The study was designed by NK and RT and supervised by RT. All authors read and agreed with the final version of the manuscript.

## Pre-publication history

The pre-publication history for this paper can be accessed here:

http://www.biomedcentral.com/1471-2407/13/575/prepub

## Supplementary Material

Additional file 1 **Localization of flotillins in MCF7 cells depleted of flotillin-1 or flotillin-2.** (A) Staining of endogenous flotillin-1 and flotillin-2 in MCF7 cells depleted of flotillin-1 (*sh*-F1-B) or flotillin-2 (*sh*-F2-B). Scale bar: 20 μm. (B) Quantification of the relative viability factor in MCF7 cells depleted of flotillin-1 (*sh*-F1-A/B) or flotillin-2 (*sh*-F2-A/B). Bars represent the mean ± SD of four independent experiments. Statistical analysis using one-way ANOVA. **, p < 0.01; ***, p < 0.001. (C) Real-time PCR data showing the relative cyclin D mRNA level in MCF7 control and MCF7 flotillin-1 (*sh*-F1-A/B) or flotillin-2 (*sh*-F2-A/B) knockdown cells. The relative expression was normalized to GAPDH and Rpl13a. Bars represent the mean ± SD of four independent experiments. Statistical analysis using one-way ANOVA. **, p < 0.01. Click here for file

Additional file 2 **EGF stimulation of MCF7 cells depleted of flotillin-1 or flotillin-2.** (A) Western blot for pY1173-EGFR, EGFR, pS473-AKT, AKT, pS338-Raf1, Raf1, pMEK1/2, MEK1/2, pERK1/2, ERK1/2, flotillin-1, flotillin-2 and GAPDH in MCF7 cells depleted of flotillin-1 (*sh*-F1-A) or flotillin-2 (*sh*-F2-B) after EGF stimulation (100 ng/ml) for 10 and 30 min. (B-G) Densitometric quantification EGFR (B), pY1173-EGFR (C, D), pMEK1/2 (E), pERK1/2 (F) and pS338-Raf1 (G). The signals of total proteins were normalized to GAPDH, phosphorylated proteins to the corresponding total protein. Bars represent the mean ± SD of four independent experiments. Statistical analysis using two-way ANOVA. *, p < 0.05; **. Click here for file

Additional file 3 **Quantitative real-time PCR analysis of EGFR expression upon PI3K inhibition.** Quantitative real-time PCR analysis showing the relative mRNA level of EGFR in MCF7 control and flotillin-1 (*sh*-F1-A/B) or flotillin-2 (*sh*-F2-A/B) knockdown cells upon PI3 kinase inhibition. The expression was normalized to GAPDH and Rpl13a. Bars represent the mean ± SD of four independent experiments. Statistical analysis using one-way ANOVA. Click here for file
