# Supplementary figures and images for: Phosphatidylinositol 3-Kinase dependent upregulation of the epidermal growth factor receptor upon Flotillin-1 depletion in breast cancer cells
Source: BMC Cancer. 2013 Dec 5;13:575. doi: 10.1186/1471-2407-13-575 (PMC4235219; doi:10.1186/1471-2407-13-575)

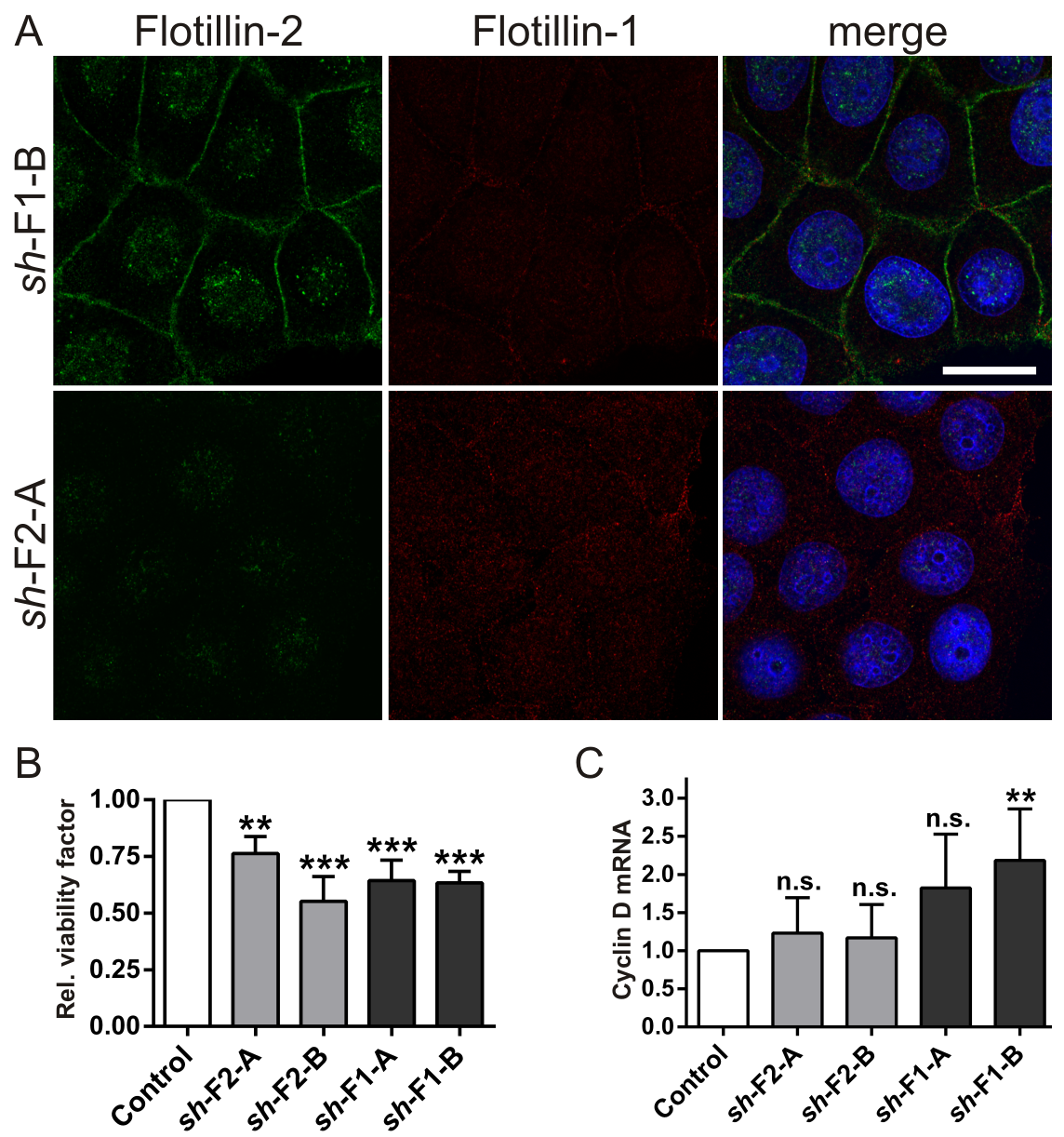

Supplement: Additional file 1 — Localization of flotillins in MCF7 cells depleted of flotillin-1 or flotillin-2. (A) Staining of endogenous flotillin-1 and flotillin-2 in MCF7 cells depleted of flotillin-1 (sh-F1-B) or flotillin-2 (sh-F2-B). Scale bar: 20 μm. (B) Quantification of the relative viability factor in MCF7 cells depleted of flotillin-1 (sh-F1-A/B) or flotillin-2 (sh-F2-A/B). Bars represent the mean ± SD of four independent experiments. Statistical analysis using one-way ANOVA. **, p < 0.01; ***, p < 0.001. (C) Real-time PCR data showing the relative cyclin D mRNA level in MCF7 control and MCF7 flotillin-1 (sh-F1-A/B) or flotillin-2 (sh-F2-A/B) knockdown cells. The relative expression was normalized to GAPDH and Rpl13a. Bars represent the mean ± SD of four independent experiments. Statistical analysis using one-way ANOVA. **, p < 0.01. [file 1471-2407-13-575-S1.tiff]

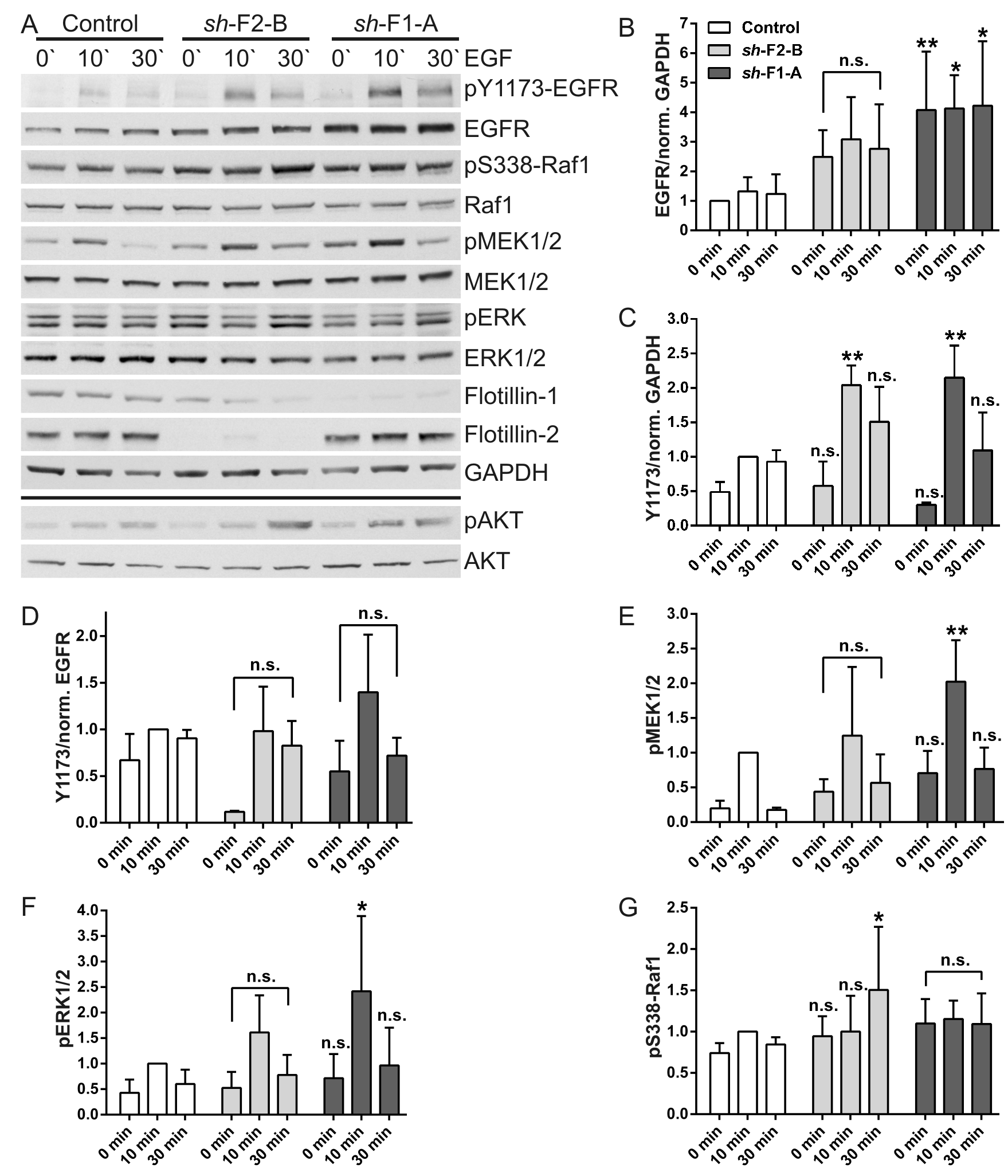

Supplement: Additional file 2 — EGF stimulation of MCF7 cells depleted of flotillin-1 or flotillin-2. (A) Western blot for pY1173-EGFR, EGFR, pS473-AKT, AKT, pS338-Raf1, Raf1, pMEK1/2, MEK1/2, pERK1/2, ERK1/2, flotillin-1, flotillin-2 and GAPDH in MCF7 cells depleted of flotillin-1 (sh-F1-A) or flotillin-2 (sh-F2-B) after EGF stimulation (100 ng/ml) for 10 and 30 min. (B-G) Densitometric quantification EGFR (B), pY1173-EGFR (C, D), pMEK1/2 (E), pERK1/2 (F) and pS338-Raf1 (G). The signals of total proteins were normalized to GAPDH, phosphorylated proteins to the corresponding total protein. Bars represent the mean ± SD of four independent experiments. Statistical analysis using two-way ANOVA. *, p < 0.05; **. [file 1471-2407-13-575-S2.tiff]

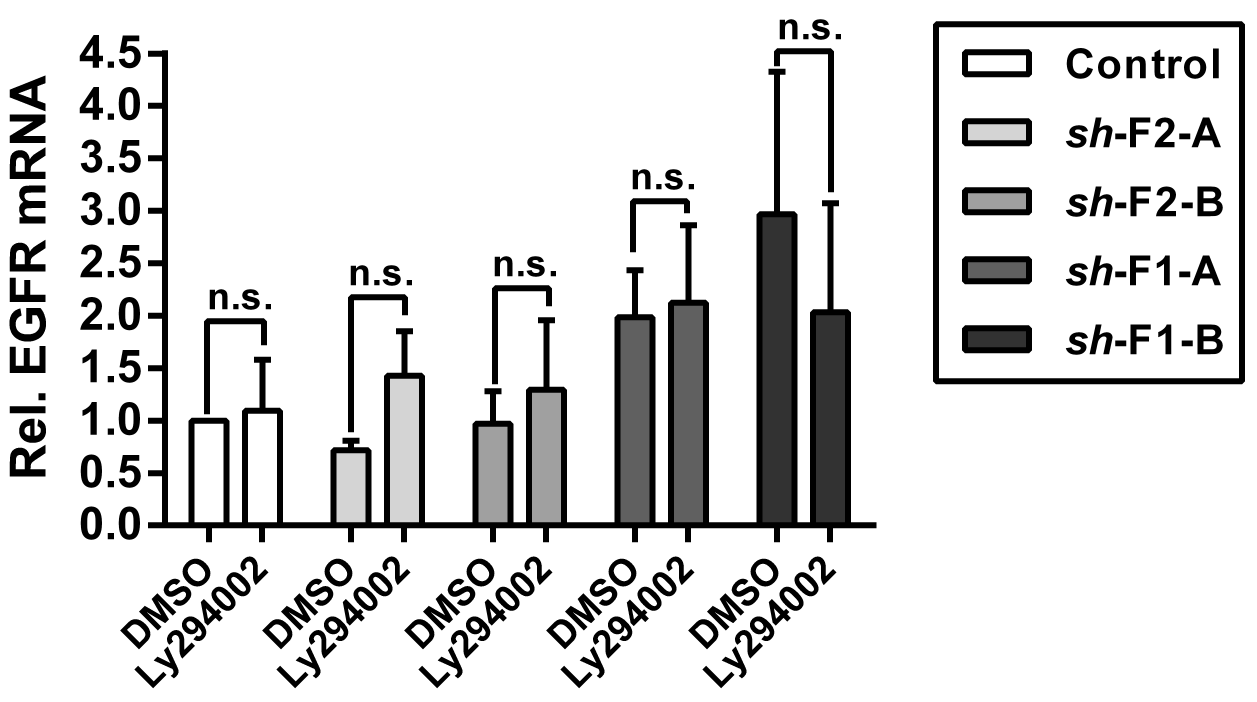

Supplement: Additional file 3 — Quantitative real-time PCR analysis of EGFR expression upon PI3K inhibition. Quantitative real-time PCR analysis showing the relative mRNA level of EGFR in MCF7 control and flotillin-1 (sh-F1-A/B) or flotillin-2 (sh-F2-A/B) knockdown cells upon PI3 kinase inhibition. The expression was normalized to GAPDH and Rpl13a. Bars represent the mean ± SD of four independent experiments. Statistical analysis using one-way ANOVA. [file 1471-2407-13-575-S3.tiff]
